# Reducing In-Hospital and 60-Day Mortality in Critically Ill Patients after Surgery with Strict Nutritional Supplementation: A Prospective, Single-Labeled, Randomized Controlled Trial

**DOI:** 10.3390/nu15214684

**Published:** 2023-11-05

**Authors:** Kyoung Moo Im, Eun Young Kim

**Affiliations:** 1 Department of Surgery, Seoul St. Mary’s Hospital, College of Medicine, The Catholic University of Korea, Banpo-daero 222, Seocho-gu, Seoul 06591, Republic of Korea; nicholas_im@naver.com; 2 Department of Surgery, Division of Trauma and Surgical Critical Care, Seoul St. Mary’s Hospital, College of Medicine, The Catholic University of Korea, Banpo-daero 222, Seocho-gu, Seoul 06591, Republic of Korea

**Keywords:** abdominal surgery, critical illnesses, NUTRIC score, nutritional support, nutrition therapy, malnutrition

## Abstract

Malnutrition in critically ill patients is a global concern, especially those who undergo abdominal surgery, as it is associated to higher infectious complications, prolonged hospital stays, and increased morbidity. Despite the importance of proper nutrition, guidelines remain broad, and practical implementation is often inadequate. We aimed to assess the effects of strict nutritional provision and investigate the appropriate target for nutrition support. A prospective, randomized controlled trial was conducted in critically ill patients admitted to intensive care units following abdominal surgery. The intervention group received targeted protein and calories, with consultation from a nutritional support team upon admission. In total, 181 patients in the intervention and 144 in the control group were analyzed. The intervention group demonstrated improved nutrition provision and subsequently better clinical outcomes, including a reduced 60-day mortality (4.4 versus 15.3, *p* = 0.001), postoperative complications (24.9 versus 47.2, *p* < 0.001), and in-hospital mortality (5 versus 17.4, *p* < 0.001). High modified nutrition risk in the critically ill scores [odds ratio (OR) = 2.658, 95% CI = 1.498–4.716] were associated with increased 60-day mortality, while active nutritional intervention (OR = 0.312, 95% CI = 0.111–0.873) was associated with lower mortality rates. Notably, the provision of targeted energy and protein alone did not exhibit a significant association with mortality outcomes.

## 1. Introduction

During the acute phase of critical illness, patients experience metabolic and physiological changes that affects their nutrition status [1,2,3]. One prominent feature is the activation of stress hormones and inflammatory mediators, which contribute to negative nitrogen balance, increased gluconeogenesis, and accelerated muscle proteolysis [1,4]. Among these patients, those who undergo abdominal surgery are particularly vulnerable to malnutrition as they experience alterations in the structural barrier of the gastrointestinal tract, impaired nutrient absorption, and prolonged fasting due to concerns such as the integrity of the anastomosis [5,6,7,8,9]. Consequently, appropriate nutritional therapy should be prioritized for critically ill patients following abdominal surgery, and it should include adequate nutritional support to preserve lean body mass and organ function [10,11].

Despite the importance of nutritional supply, the recommendations for protein or calorie intake vary according to different guidelines [2,4,12], and this is the same for surgical patients [2,12,13]. Additionally, patients often experience a delay in initiating nutritional support, and several studies reported that in clinical practice only 39–63% of the intended energy and 45–55% of the prescribed protein are being administered to critically ill patients during the acute phase [14,15,16,17,18]. Furthermore, recent randomized controlled trials reported conflicting results with current guidelines, with some suggesting that lower calorie or higher protein administration did not significantly impact clinical outcomes and may even worsen the outcomes for certain group of patients [16,19]. Thus, the optimal nutritional provision target during the acute phase of critical illness, particularly for surgical patients, remains controversial, and there is no standardized protocol.

In our previous study, the malnutrition status upon admission, indicated by a modified nutrition risk in the critically ill (mNUTRIC) score of five or higher, and a low energy adequacy during intensive care unit (ICU) stay were revealed as predictors for mortality in critically ill patients following abdominal surgery [20]. We aimed to assess the effects of strict nutritional provision, targeting an energy adequacy of 80% or more and a protein intake of at least 1.5 g/kg/day, on in-hospital and 60-day mortality. Additionally, we investigate the appropriate target for nutrition support in critically ill patients who undergo abdominal surgery.

## 2. Participants and Methods

### 2.1. Patient Enrollment and Exclusion Criteria

Patients admitted to our institution’s surgical ICU immediately after abdominal surgery from March 2019 to August 2022 were eligible for study enrollment. All patients who underwent abdominal surgery within the department of general surgery were considered for inclusion, spanning across various subspecialties such as hepatobiliary, pancreatic, gastrointestinal, colorectal, vascular, and trauma surgery. They were enrolled regardless of the surgical method, either open, laparoscopy, or robotic. The exclusion criteria were as follows; (1) aged under 18 years, (2) underwent surgery under local or regional anesthesia, (3) pregnant, (4) readmitted to the ICU, (5) diagnosed with renal failure and receiving renal replacement therapy, (6) lacked individual data necessary to calculate the mNUTRIC score measured at ICU admission, (7) failed to provide informed consent, or (8) with ‘do-not-resuscitate’ status. If the patient was discharged or expired within 48 h of ICU admission, the case was excluded from the analysis. Patients diagnosed with multiorgan failure, represented by a high sequential organ failure assessment (SOFA) score (≥9) upon ICU admission, were also excluded from the results analysis. The current study was approved and carefully monitored by the Institutional Review Board of the Ethics Committee at our institution (IRB No. KC23RISI0361). Informed consent was obtained from all individual participants or participant guardians, and the trial was conducted according to the Declaration of Helsinki and its later amendments. The trial is registered at Clinicals.gov (NCT06058247).

### 2.2. Sample Size Calculation

The sample size was estimated based on the results of previous studies in which the 60-day mortality in a high-protein group (≥1.2 g/kg/day) and control group was 11% and 43%, respectively [21], and this indicated that more than 144 patients per group would achieve 90% power at a two-tailed α of 0.05 for a 17.7% reduction in 60-day mortality. Finally, we set the number of enrolled patients to 181 or more per group, considering a dropout rate of 20%.

### 2.3. Randomization and the Study Protocol

Randomization in permuted blocks of two and an allocation ratio of 1:1 was performed for patient allocation using a computerized automated system. The recruited participants were randomly assigned to one of two groups and were not aware of their assigned randomization group. The intervention group received consultation from the nutritional support team (NST) upon ICU admission, and nutritional supplementation was initiated on the same day. The NST is a multidisciplinary support team comprised of physicians, nurses, dietitians, and pharmacists, which assesses the nutritional status of patients, determines their nutritional needs, and provides recommendations for nutritional therapy. The targets in the intervention group were protein supplementation at over 1.5 g/kg/day, calorie provision at over 20 kcal/kg/day, and energy adequacy of at least 80%. The energy target was estimated by multiplying the resting energy expenditure using the Harris and Benedict equation by an activity factor of 1.3 and a stress factor of 1.1 [22]. Actual body weight was used as the body weight for patients with a percent of ideal body weight (PIBW) of less than 120%, while adjusted body weight was used for patients with a PIBW greater than or equal to 120%. The control group received the ‘usual care’ that included a conservative nutritional management without specific protein or caloric target, and a volume-based feeding protocol with stomach feeding in the same way as in the previous study [20]. Gastric residual volumes were measured every 6 h, and if the residual volume was higher than 500 mL/6 h, the attending physician may have delayed the enteral feeding. However, in this situation, we also examined the abdomen for intolerance, and when there was no sign of acute abdominal complications, we usually applied prokinetics or other medication rather than stopping the feeding. The average daily protein and caloric intake during ICU admission were used as early protein and caloric intake, respectively. Parenteral nutrition (PN) was supplied as a total nutritional admixture, including glucose, amino acids, and lipids. However, if the triglyceride level exceeded 400 mg/dL or liver function was impaired, lipid-free PN was administered. In terms of the nutrient supply route, a strategy to attain the target nutritional value was tailored for each patient in consultation with the NST, relying on continuous nutritional assessments throughout their ICU stay. For oral supplementation, we provided NUCARE^®^ (Miwon Co., Ltd., Anyang-si, Republic of Korea) or Encover (JW Pharmaceutical, Seoul, Republic of Korea), both of which are ready-made liquid products available in both can and ready-to-hang tube feeding bag formula. These products provide a balanced mix of energy, protein, and essential micronutrients. In 100 mL of NUCARE^®^ (Miwon Co., Ltd.) or Encover (JW Pharmaceutical), 14–15 g of carbohydrates, 4–4.4 g of proteins, and 2–3 g of lipids were included. Also, 100 kcal of total calories would be supplied. It included various vitamins and minerals with added arginine and omega-6 and omega-3 (the omega-6 to omega-3 ratio was 3:1). For the parenteral nutrition support, OLIMEL^®^ N9E (Baxter, Glenview, IL, USA) was served as the parenteral nutrition product. In 1000 mL of OLIMEL^®^ N9E (Baxter), 110 g of glucose, 56 g of amino acids, 9 g of nitrogen, and 40 g of lipids were included. Also, 1070 kcal of total calories would be supplied. It included refined olive oil and soya-bean oil, with added arginine, glutamine, and various vitamins and minerals. In both the intervention and control groups, we ensured the addition of trace elements and vitamins in appropriate amounts to meet the specific requirements of each patient. The CONSORT 2010 checklist of information of current trial can be downloaded at the Supplementary Materials.

### 2.4. Data Collection and Outcome Measurement

The data were obtained from the electronic medical records, operative reports, and nursing charts. The collected data included demographics and the laboratory profiles of nutritional status such as total protein, albumin, prealbumin, transferrin, and cholesterol levels at the time of admission to the ICU after surgery. SOFA and mNUTRIC scores were also calculated at the time of ICU admission and recorded as well. The mNUTRIC score was calculated from five variables that included age, the acute physiology and chronic health evaluation (APACHE) II score, the SOFA score, the number of comorbidities, and the days from hospital to ICU admission, as described previously by Rahman et al. [23]. The total mNUTRIC scores ranged from 0 to 9 points. All participants in the intervention group and control group were subcategorized into high and low mNUTRIC groups using an appropriate mNUTRIC cut-off score of 5 as described in a previous report to predict 90-day mortality for further analysis [20]. Daily nutritional delivery data were recorded for all participants, including the feeding strategy, type, and amount of nutrients received by the patients. The total daily calories and protein prescribed or delivered to each patient were calculated. We defined energy adequacy (%) as the total calories delivered divided by the total calories prescribed multiplied by 100.

Postoperative complications of Grade III or more, according to the Clavien–Dindo classification, were analyzed [24]. Grade III complications were any cases requiring surgical, endoscopic, or radiological interventions, Grade IV complications were cases showing life-threatening morbidities, and Grade V complications were defined as the death of a patient. Any cases that needed medical intervention, such as renal replacement therapy, mechanical ventilator, or extracorporeal membrane oxygenation, during the ICU stay after surgery were recorded. In-hospital mortality was defined as the occurrence of death during the same hospitalization period. The 60-day mortality was defined as any mortality that developed within 60 days after surgery, whether as an inpatient or outpatient. Overall, mortality was any mortality that developed during the study period.

### 2.5. Study Outcomes and Statistical Analysis

The primary outcome of the current study was the 60-day mortality rate after surgery in the two groups classified according to whether or not the nutritional intervention was implemented. The secondary outcome was the in-hospital mortality rate and the incidence of postoperative morbidities in the two groups. Continuous data are expressed as the mean ± standard deviation and analyzed using Student’s *t*-test. Variables were tested for normal distribution using the Kolmogorov–Smirnov test, and in the case of variables not normally distributed, the Mann–Whitney test was used. Categorical data are presented as proportions and analyzed using the Chi-square test or Fisher’s exact test. In order to identify predisposing factors of clinical outcomes such as postoperative complications, in-hospital mortality, and 60-day mortality, only significant variables in univariate analysis defined as a case with a *p*-value < 0.05 were used in multivariate analysis using Cox’s proportional hazard model. The hazard ratio was expressed as the relative risk with corresponding 95% confidence intervals (CI). All analyses were performed with the SPSS statistical package software for Windows (version 24.0; SPSS Inc., Chicago, IL, USA). Statistical tests were conducted two-sided, and a *p*-value < 0.05 was considered statistically significant.

## 3. Results

**Figure 1:**
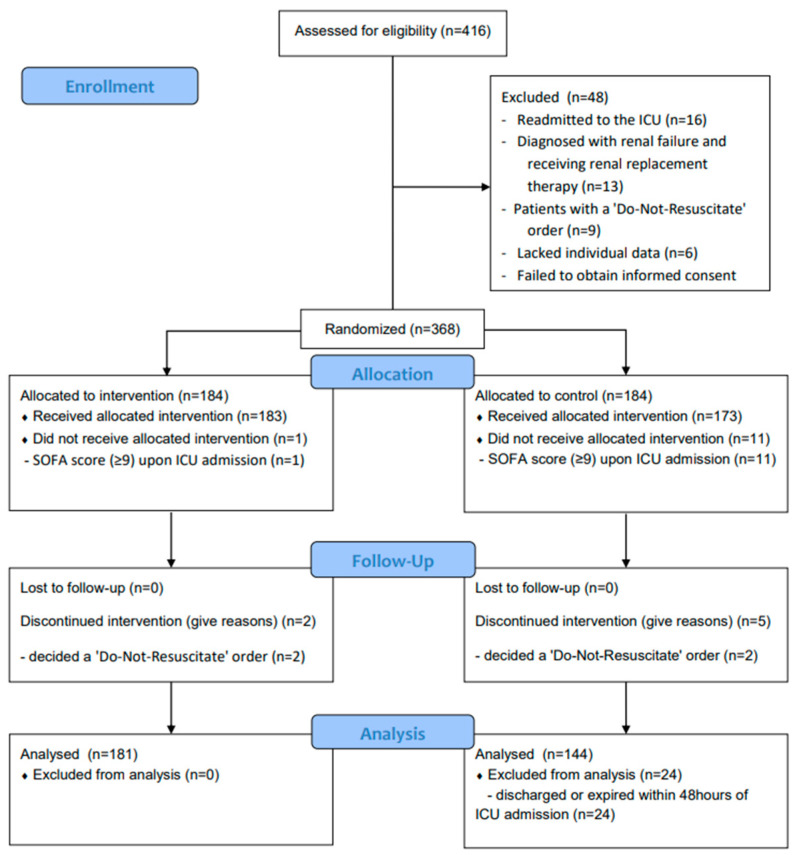
Flow chart of study participants.

A total of 416 patients were eligible for study enrollment between March 2019 and August 2022. According to our study criteria, 48 patients were excluded from enrollment, and a total of 368 patients were enrolled in the current study. The patients were randomly assigned to 184 patients in the intervention group and 184 patients in the control group. Of these, 43 patients dropped out during the study period or were excluded from the analysis (3 patients in the intervention group and 40 patients in the control group). The final analysis included 181 patients in the intervention group and 144 patients in the control group. The schematic diagram of study enrollment is shown in Figure 1.

A comparison of the demographics and clinical outcomes of the study participants is presented in Table 1. The control group showed a significantly lower mean age and higher SOFA and APACHE II scores compared to the intervention group. There were no significant differences in nutritional assessment scores calculated by mNUTRIC, SGA, and body mass index (BMI) between the two groups. In clinical outcomes, the intervention group showed significantly lower rates of postoperative complications, in-hospital mortality, 60-day mortality, and overall mortality compared to the control group. Table 2 presents the results of the comparative analysis of characteristics and clinical outcomes after subgroup analysis, focusing on nutritionally high-risk patients. The mean age was higher in the intervention group, while the other characteristics were not significantly different between the two groups. In the clinical outcomes, the rate of in-hospital mortality, 60-day mortality, and overall mortality was significantly lower in the intervention group compared to the control group.

Regarding the assessment of nutritional provision (Table 3), the intervention group received an average energy intake of 22.0 ± 7.4 kcal/kg/day and a protein intake of 1.17 ± 0.43 g/kg/day. In contrast, the control group received an average energy intake of 10.5 ± 5.2 kcal/kg/day and protein intake of 0.49 ± 0.25 g/kg/day. The energy adequacy in the intervention group was significantly higher than that of the control group, with rates of 80.7% and 42.2%, respectively.

Table 4 presents the logistic regression analysis results aimed at identifying the predictors of postoperative complications. High SOFA and mNUTRIC scores were associated with higher mortality rates in the total participant group (Table 4A). The odds ratio for protein provision showed that a protein supply of ≥1.2 g/kg/day was significantly associated with a lower risk of postoperative complications. For nutritionally high-risk patients (mNUTRIC scores ≥ 5), (Table 4B) univariate analysis identified age, SOFA score, mNUTRIC score, emergent surgery, average energy intake of ≥20 kcal/kg/day, and protein intake ≥ 1.2 g/kg/day as factors significantly associated with postoperative complications. However, none of the variables showed significant associations with postoperative complications in the multivariate analysis. The cut-off energy and protein intake values were determined based on the actual nutrients delivered in the intervention group (Table 3). The appropriateness of these cut-off values was validated using receiver operating characteristic analysis.

Table 5 and Table 6 present the results of logistic regression analysis for identifying the risk factors for in-hospital mortality and 60-day mortality after surgery, respectively. Multivariate analysis revealed that high mNUTRIC scores and emergent surgery were associated with a higher in-hospital mortality, whereas the implementation of active nutritional interventions, as performed in this study was associated with a significantly lower in-hospital mortality (Table 5). Regarding 60-day mortality after surgery, high mNUTRIC scores, emergent surgery, and the implementation of active nutritional intervention in the early postoperative period were associated with 60-day mortality in both the total participants and the nutritionally high-risk patients (Table 6).

## 4. Discussion

In the current study, the intervention group with active nutritional provision received nutritional support closer to the targeted goals and exhibited better clinical outcomes, including postoperative complications and mortality. High mNUTRIC scores were associated with higher mortality rates, whereas the active nutritional intervention was linked to lower mortality rates. However, the implementation status of either energy or protein intervention alone was not significantly associated with mortality outcomes.

Postoperative patients undergo a state of significant stress, which has the potential to profoundly affect their physiologic responses [4,25]. The activation of stress hormones as part of the systemic inflammatory response syndrome and cytokine release [1,10,26,27] led to various metabolic changes, such as accelerated protein breakdown, hypermetabolism, increased lipolysis, elevated endogenous hepatic glucose production, and reduced glucose clearance [4,10]. As surgical procedures induce these responses, they ultimately make patients more susceptible to malnutrition, and the magnitude of these metabolic changes is closely related to the severity and extent of the surgical injury [13]. Thus, critically ill patients undergoing major surgery can easily become malnourished, predisposing them to increased postoperative morbidity and mortality [28]. In addition to these factors, there is a lack of high-quality evidence in this field, and the recommended values often vary widely. For instance, in the practical guidelines of the United States [12] and Europe [2,4], protein intake for critically ill patients ranges from 1.2 to 2.0 g/kg/day or an equivalent of 1.3 g/kg/day. For surgical patients, it is suggested to provide an additional 15–30 g of protein per liter of lost exudate or a fixed amount of 1.5 g/kg/day, respectively. Regarding calorie intake, the target recommendation for critically ill and surgical patients in the United States is 25–30 kcal/kg/day, whereas, in the European guidelines, it is recommended to provide 20–25 kcal/kg/day for critically ill patients and 25–30 kcal/kg/day for surgical patients. In the current study, we targeted a protein intake of 1.5 g/kg/day and a caloric intake of 20 kcal/kg/day or more for the intervention group, and significantly lower rates of in-hospital mortality and 60-day mortality were seen in the intervention group. In the subgroup analysis focusing on high mNUTRIC scores of five or higher, the intervention group still showed a significantly lower in-hospital and 60-day mortality. These results suggest an association between strict nutritional intervention and improved clinical outcomes. The multivariate analysis results indicated that while no significant association was found between mortality and the delivery of an average energy of ≥20 kcal/kg/day or protein of ≥1.2 g/kg/day, the intervention was significantly associated with reduced in-hospital mortality and 60-day mortality. Previous studies by Bargetzi et al. [29] and Richards et al. [30] reported that the implementation of individualized and early nutritional support aimed at achieving nutritional goals was associated with an improvement in clinical outcomes. In our study, factors such as nutritional assessment and early nutritional provisioning after ICU admission were suspected to have an impact on mortality in the intervention group [3,29,30]. These findings may underscore the significance of comprehensive nutritional management, particularly for surgical patients at a high nutritional risk.

Additionally, regarding the actual nutrient provision to patients based on interventions, the control group received less than half of the targeted energy and protein, whereas the intervention group received an average energy of 22 kcal/kg/day and protein of 1.17 g/kg/day. The findings from the 2014 International Nutrition Survey from 187 ICUs worldwide revealed that ICU patients received only 62% of the prescribed calories and 55% of the prescribed protein [15,18]. Our results showed similar, larger gaps between prescribed nutrition and actual delivery to patients in the control group. The multidisciplinary approach through NST combined with early and active nutrition intervention immediately after surgery is likely to have contributed to the observed outcomes in the intervention group. The findings highlight the importance of continuous attention and monitoring with active nutritional intervention to ensure that critically ill patients receive the amount of nutrition they require.

Another interesting finding of the study was the significance of mNUTRIC scores as a predisposing factor for both in-hospital and 60-day mortality after surgery. Various nutritional screening and assessment tools have been used for critically ill patients. The American Society for Parenteral and Enteral Nutrition guidelines suggest the use of nutritional risk screening (NRS) 2002 or NUTRIC scores to determine nutritional risk, while the European Society of Clinical Nutrition and Metabolism guideline disagrees with categorizing patients according to NRS 2002 or NUTRIC scores to define their nutritional regimen due to the lack of a gold standard for identifying at-risk patients [2,12]. However, it is important to consider that body weight fluctuations can occur in critically ill patients who have undergone abdominal surgery due to factors such as critical illness-related edema or resuscitation during surgery. Therefore, NRS 2002, which includes BMI measurements, may yield inaccurate results [31]. Although there has been limited widespread use and a lack of supporting evidence to date, our results suggest that mNUTRIC scores can be a useful tool for assessing nutritional risk in critically ill surgical patients following abdominal surgery. The advantage of this scoring system is that it can easily assess both nutrition status and disease severity upon ICU admission without the need to measure special markers such as interleukin-6 levels [23,32,33].

Despite our interesting findings, it is important to interpret the results with caution due to certain limitations. Firstly, there was an uneven dropout rate between the two groups, resulting in imbalances in some baseline variables such as age, gender, and SOFA score. These imbalances may have introduced bias into the estimates or influenced the results. However, it is worth noting that all baseline nutritional status measurements, including mNUTRIC scores, SGA, and BMI, were balanced between the two groups, which helped to mitigate some of the potentially confounding effects. Additionally, imbalanced characteristics related to host fragility or disease severity, such as age or SOFA scores, were found to be more unfavorable in the intervention group. However, our results showed that the clinical outcomes were superior in the intervention group. Thus, it can be expected that the impact of confounding due to these baseline differences on the final result would be insignificant. Secondly, caloric prescriptions in the current study were determined using weight-based formulas rather than indirect calorimetry, which may pose a risk of overfeeding or underfeeding. However, considering that most surgical patients were not on mechanical ventilation, the use of indirect calorimetry for these patients may have been limited. Lastly, our study enrolled a small number of patients in a single institution, limiting the generalizability of the results. Further research with a larger group of patients and multicenter studies are needed to address these limitations and provide more comprehensive guidelines on the nutritional management of critically ill patients following abdominal surgery.

## 5. Conclusions

Our study highlights the importance of comprehensive nutritional management for surgical patients, especially those at a high nutritional risk. The intervention group, targeted with specific nutritional goals, showed better clinical outcomes of postoperative complications and mortality. High mNUTRIC scores were associated with higher mortality, whereas the active nutritional intervention was linked to lower mortality. These findings emphasize the need for early and individualized nutritional support to improve patient outcomes.

## Figures and Tables

**Table 1:** Characteristics of the studied population and differences in clinical characteristics and initial laboratory findings between the intervention and control group.

Variables	All Patients	Intervention Group	Control Group	*p*-Value
	*n = 325*	*n = 181*	*n = 144*	
**Demographics**				
Age (years)	65 ± 14.6	67.9 ± 13.6	61.3 ± 15.1	*<0.001*
Gender (male, %)	217 (66.8)	112 (61.9)	105 (72.9)	*0.044*
Body mass index (kg/m^−2^)	23.5 ± 4.1	23.3 ± 4.1	23.7 ± 4.2	*0.309*
Use of vasopressors (%)	75 (23.1)	42 (56)	33 (22.9)	*1.000*
SOFA score	5.1 ± 3.5	4.8 ± 3.5	5.6 ± 3.5	*0.041*
APACHE II score	14.1 ± 7.2	12.5 ± 6.5	16 ± 7.5	*<0.001*
mNUTRIC score	3.8 ± 1.8	3.9 ± 1.8	3.8 ± 1.7	*0.640*
Patients with high mNUTRIC score ≥ 5	108 (27)	65 (25.4)	43 (29.9)	*0.349*
SGA (%)				*0.409*
well-nourished	234 (72)	131 (72.4)	103 (71.5)	*0.901*
moderately malnourished	60 (18.5)	30 (16.6)	30 (20.8)	*0.388*
severely malnourished	31 (9.5)	20 (11)	11 (7.6)	*0.345*
**Clinical outcomes**				
Postoperative complication (%)	113 (34.8)	45 (24.9)	68 (47.2)	*<0.001*
Length of ICU stay (days)	5.5 ± 6.2	5 ± 5.7	6.1 ± 6.8	*0.115*
Length of hospital stay (days)	28.6 ± 23.9	23.5 ± 20.5	35.1 ± 26.2	*<0.001*
In-hospital mortality (%)	34 (10.5)	9 (5)	25 (17.4)	*<0.001*
60-day mortality (%)	30 (9.2)	8 (4.4)	22 (15.3)	*0.001*
Overall mortality (%)	36 (11.1)	9 (5)	27 (18.8)	*<0.001*
**Laboratory test**				
Total protein (g/dL)	5.5 ± 1.2	5.7 ± 1.3	5.3 ± 1.1	*0.005*
Albumin (g/dL)	3.2 ± 0.9	3.2 ± 1.1	3.2 ± 0.6	*0.965*
Prealbumin (mg/dL)	15.7 ± 7.6	15.9 ± 7.3	15 ± 8.4	*0.435*
Transferrin (mg/dL)	154.9 ± 59.7	163.3 ± 60.8	140.1 ± 55	*0.040*
Total cholesterol (mg/dL)	105.5 ± 43.2	107.7 ± 43	101.8 ± 43.4	*0.303*
Triglycerides (mg/dL)	75.3 ± 51.5	73.6 ± 47.8	78.1 ± 57.4	*0.519*
HDL (mg/dL)	28.8 ± 13.3	30.4 ± 13.8	26.2 ± 12.1	*0.019*
LDL (mg/dL)	55.8 ± 29.5	56.6 ± 31.6	54.3 ± 25.7	*0.571*

APACHE, acute physiology and chronic health evaluation; HDL, high-density lipoproteins; ICU, intensive care unit; LDL, low-density lipoproteins; mNUTRIC, modified nutrition risk in critically ill; SGA, subjective global assessment; SOFA, sequential organ failure assessment score.

**Table 2:** Characteristics and clinical outcomes of patients with the high mNUTRIC score (mNUTRIC ≥ 5) in enrolled participants.

Variables	All Patients	Intervention Group	Control Group	*p*-Value
	*n = 108*	*n = 65*	*n = 43*	
**Demographics**				
Age (years)	70.6 ± 12.8	72.7 ± 12.2	67.4 ± 13.2	*0.034*
Gender (male, %)	69 (63.9)	41 (63.1)	28 (65.1)	*1.000*
Body mass index (kg/m^−2^)	23 ± 4.5	22.7 ± 4.6	23.4 ± 4.5	*0.466*
Use of vasopressors (%)	53 (49.1)	35 (53.8)	18 (41.9)	*0.244*
SOFA score	8.1 ± 3.3	8.2 ± 3.1	8 ± 3.5	*0.756*
APACHE II score	16.7 ± 6.7	17.1 ± 5.9	16.1 ± 7.8	*0.439*
mNUTRIC score	5.9 ± 1	6 ± 1	6 ± 1	*0.902*
Patients with high mNUTRIC score ≥ 5				*0.209*
SGA (%)	65 (60.2)	36 (55.4)	29 (67.4)	*0.234*
well-nourished	25 (23.1)	15 (23.1)	10 (23.3)	*1.000*
moderately malnourished	18 (16.7)	14 (21.5)	4 (9.3)	*0.118*
severely malnourished	70.6 ± 12.8	72.7 ± 12.2	67.4 ± 13.2	*0.034*
**Clinical outcomes**				
Postoperative complication (%)	65 (60.2)	35 (53.8)	30 (69.8)	*0.112*
Length of ICU stay (days)	8.3 ± 8.7	8.4 ± 7.8	8.3 ± 10	*0.954*
Length of hospital stay (days)	35.4 ± 23.6	33.6 ± 22.8	38.2 ± 24.7	*0.327*
In-hospital mortality (%)	21 (19.4)	8 (12.3)	13 (30.2)	*0.027*
60-day mortality (%)	19 (17.6)	7 (10.8)	12 (27.9)	*0.037*
Overall mortality (%)	21 (19.4)	8 (12.3)	13 (30.2)	*0.027*
**Laboratory test**				
Total protein (g/dL)	5 ± 1.1	4.9 ± 1.2	5 ± 1	*0.532*
Albumin (g/dL)	3 ± 1.2	2.9 ± 1.5	3.1 ± 0.5	*0.430*
Prealbumin (mg/dL)	11.3 ± 6	11.1 ± 5.4	12.2 ± 8.4	*0.591*
Transferrin (mg/dL)	122.7 ± 43.2	123.7 ± 47.8	120.4 ± 30.3	*0.737*
Total cholesterol (mg/dL)	83.9 ± 33.7	82.5 ± 34.3	88.6 ± 32.9	*0.636*
Triglycerides (mg/dL)	70.6 ± 50.3	68.3 ± 49.9	75.4 ± 52.1	*0.584*
HDL (mg/dL)	23.4 ± 12.9	24.1 ± 14.1	21.9 ± 10.1	*0.469*
LDL (mg/dL)	40.8 ± 21.8	38.6 ± 22.5	45.4 ± 19.9	*0.225*

APACHE, acute physiology and chronic health evaluation; HDL, high-density lipoproteins; ICU, intensive care unit; LDL, low-density lipoproteins; mNUTRIC, modified nutrition risk in critically ill; SGA, subjective global assessment; SOFA, sequential organ failure assessment score.

**Table 3:** Average of calorie and protein delivered between intervention and control group in (**A**) in total participants and (**B**) in the high mNUTRIC group (mNUTRIC score ≥ 5).

(A) Total Participants				
Variables	All Patients	Intervention Group	Control Group	*p*-Value
	*n = 325*	*n = 181*	*n = 144*	
Total calorie need	1543.5 ± 233.6	1619.9 ± 257.5	1447.4 ± 153.2	*<0.001*
Energy delivered per day (kcal/day)	993.3 ± 525	1307.9 ± 456.8	597.9 ± 281.3	*<0.001*
Energy adequacy (%)	63.7 ± 29.4	80.7 ± 23	42.2 ± 21.5	*<0.001*
Average energy delivered (kcal/kg/day)	16.9 ± 8.6	22 ± 7.4	10.5 ±5.2	*<0.001*
Protein delivered per day (g/day)	51.6 ± 32.6	70.7 ± 30.5	27.6 ± 13.7	*<0.001*
Average protein delivered (g/kg/day)	0.87 ± 0.5	1.17 ± 0.43	0.49 ± 0.25	*<0.001*
**(B) High mNUTRIC Group (mNUTRIC Score ≥ 5)**				
**Variables**	**All Patients**	**Intervention Group**	**Control Group**	***p-Value***
	***n = 108***	***n = 65***	***n = 43***	
Total calorie need	1444.9 ± 188.4	1470.7 ± 204	1405.9 ± 156.2	*0.080*
Energy delivered per day (kcal/day)	877 ± 418	1033 ± 399.4	641.3 ± 327.9	*<0.001*
Energy adequacy (%)	61.7 ± 28.7	70.8 ± 25.5	48 ± 28.1	*<0.001*
Average energy delivered (kcal/kg/day)	15.8 ± 7.8	18.5 ± 7.4	11.8 ± 6.6	*<0.001*
Protein delivered per day (g/day)	43.7 ± 24.6	53.5 ± 24.3	29 ± 16.5	*<0.001*
Average protein delivered (g/kg/day)	0.78 ± 0.44	1 ± 0.4	0.5 ± 0.3	*<0.001*

**Table 4:** Univariate and multivariate analysis of risk factors for postoperative complications (**A**) in total participants and (**B**) in the high mNUTRIC group (mNUTRIC score ≥ 5).

(A) Total Participants				
Variable	Univariate		Multivariate	
	OR (95% CI)	*p*	OR (95% CI)	*p*
Age	0.989 (0.974–1.005)	*0.183*		
SOFA score	1.513 (1.376–1.664)	*<0.001*	1.338 (1.197–1.496)	*<0.001*
APACHE II score	1.086 (1.050–1.122)	*<0.001*	1.008 (0.967–1.051)	*0.708*
mNUTRIC score	1.861 (1.580–2.193)	*<0.001*	1.272 (1.044–1.549)	*0.017*
Emergent surgery	3.175 (2.044–4.933)	*<0.001*	1.726 (0.979–3.042)	*0.059*
Average energy delivered ≥ 20 (kcal/kg/day)	0.189 (0.112–0.317)	*<0.001*	1.226 (0.430–3.499)	*0.703*
Average protein delivered ≥ 1.2 (g/kg/day)	0.170 (0.104–0.278)	*<0.001*	0.303 (0.113–0.814)	*0.018*
Intervention	0.370 (0.231–0.592)	*<0.001*	0.594 (0.294–1.200)	*0.147*
**(B) High mNUTRIC Group (mNUTRIC Score ≥ 5)**				
**Variable**	**Univariate**		**Multivariate**	
	**OR (95% CI)**	***p***	**OR (95% CI)**	***p***
Age	0.958 (0.926–0.992)	*0.015*	0.976 (0.936–1.016)	*0.238*
SOFA score	1.338 (1.155–1.550)	*<0.001*	1.197 (0.991–1.447)	*0.062*
APACHE II score	0.998 (0.942–1.058)	*0.959*		
mNUTRIC score	2.223 (1.376–3.591)	*0.001*	1.660 (0.900–3.059)	*0.104*
Emergent surgery	3.175 (2.044–4.933)	*<0.001*	1.799 (0.727–4.449)	*0.204*
Average energy delivered ≥ 20 (kcal/kg/day)	0.314 (0.132–0.752)	*0.009*	0.990 (0.197–4.984)	*0.990*
Average protein delivered ≥ 1.2 (g/kg/day)	0.371 (0.164–0.838)	*0.017*	0.830 (0.179–3.844)	*0.812*
Intervention	0.506 (0.224–1.140)	*0.100*	0.468 (0.160–1.365)	*0.164*

APACHE, acute physiology and chronic health evaluation; mNUTRIC, modified nutrition risk in critically ill; SOFA, sequential organ failure assessment score.

**Table 5:** Univariate and multivariate analysis of risk factors for in-hospital mortality (**A**) in total participants and (**B**) in the high mNUTRIC group (mNUTRIC score ≥ 5).

(A) Total Participants				
Variable	Univariate		Multivariate	
	OR (95% CI)	*p*	OR (95% CI)	*p*
Age	1.010 (0.985–1.036)	*0.442*		
SOFA score	1.295 (1.167–1.437)	*<0.001*	1.119 (0.974–1.284)	*0.112*
APACHE II score	1.046 (1.004–1.090)	*0.030*	0.979 (0.928–1.033)	*0.439*
mNUTRIC score	1.733 (1.393–2.155)	*<0.001*	1.419 (1.086–1.855)	*0.010*
Emergent surgery	5.541 (2.614–11.743)	*<0.001*	3.842 (1.691–8.725)	*0.001*
Average energy delivered ≥ 20 (kcal/kg/day)	0.223 (0.091–0.546)	*0.001*	1.256 (0.286–5.520)	*0.763*
Average protein delivered ≥ 1.2 (g/kg/day)	0.235 (0.105–0.525)	*<0.001*	0.619 (0.166–2.306)	*0.475*
Intervention	0.249 (0.112–0.553)	*0.001*	0.300 (0.122–0.739)	*0.009*
**(B) High mNUTRIC Group (mNUTRIC Score ≥ 5)**				
**Variable**	**Univariate**		**Multivariate**	
	**OR (95% CI)**	***p***	**OR (95% CI)**	***p***
Age	1.009 (0.971–1.049)	*0.634*		
SOFA score	1.183 (1.004–1.393)	*0.044*	1.017 (0.832–1.243)	*0.870*
APACHE II score	0.991 (0.921–1.065)	*0.796*		
mNUTRIC score	2.483 (1.444–4.272)	*0.001*	2.792 (1.340–5.817)	*0.006*
Emergent surgery	3.872 (1.204–12.458)	*0.023*	4.563 (1.207–17.250)	*0.025*
Average energy delivered ≥ 20 (kcal/kg/day)	0.370 (0.101–1.364)	*0.135*		
Average protein delivered ≥ 1.2 (g/kg/day)	0.367 (0.114–1.183)	*0.093*		
Intervention	0.324 (0.121–0.868)	*0.025*	0.197 (0.059–0.657)	*0.008*

APACHE, acute physiology and chronic health evaluation; mNUTRIC, modified nutrition risk in critically ill; SOFA, sequential organ failure assessment score.

**Table 6:** Univariate and multivariate analysis of risk factors for 60-day mortality (**A**) in total participants and (**B**) in the high mNUTRIC group (mNUTRIC score ≥ 5).

(A) Total Participants				
Variable	Univariate		Multivariate	
	OR (95% CI)	*p*	OR (95% CI)	*p*
Age	1.008 (0.982–1.035)	*0.556*		
SOFA score	1.258 (1.131–1.399)	*<0.001*	1.063 (0.921–1.227)	*0.406*
APACHE II score	1.045 (1.001–1.091)	*0.046*	0.981 (0.927–1.039)	*0.514*
mNUTRIC score	1.771 (1.405–2.234)	*<0.001*	1.538 (1.154–2.052)	*0.003*
Emergent surgery	5.547 (2.304–13.353)	*<0.001*	3.575 (1.510–8.465)	*0.004*
Average energy delivered ≥ 20 (kcal/kg/day)	0.215 (0.081–0.567)	*0.002*	2.278 (0.406–12.775)	*0.349*
Average protein delivered ≥ 1.2 (g/kg/day)	0.156 (0.059–0.413)	*<0.001*	0.228 (0.047–1.119)	*0.069*
Intervention	0.256 (0.111–0.595)	*0.002*	0.351 (0.138–0.897)	*0.029*
**(B) High mNUTRIC Group (mNUTRIC Score ≥ 5)**				
**Variable**	**Univariate**		**Multivariate**	
	**OR (95% CI)**	***p***	**OR (95% CI)**	***p***
Age	1.000 (0.962–1.040)	*0.981*		
SOFA score	1.167 (0.987–1.381)	*0.071*		
APACHE II score	0.993 (0.921–1.070)	*0.857*		
mNUTRIC score	2.658 (1.498–4.716)	*0.001*	3.106 (1.532–6.298)	*0.002*
Emergent surgery	3.271 (1.005–10.644)	*0.049*	4.242 (1.054–17.076)	*0.042*
Average energy delivered ≥ 20 (kcal/kg/day)	0.256 (0.066–1.186)	*0.082*		
Average protein delivered ≥ 1.2 (g/kg/day)	0.173 (0.038–0.796)	*0.024*	0.233 (0.040–1.369)	*0.107*
Intervention	0.312 (0.111–0.873)	*0.026*	0.254 (0.070–0.918)	*0.037*

APACHE, acute physiology and chronic health evaluation; mNUTRIC, modified nutrition risk in critically ill; SOFA, sequential organ failure assessment score.

## Data Availability

The datasets used and/or analyzed during the current study are available from the corresponding author on reasonable request.

## Supplementary Materials

The following supporting information can be downloaded at: https://www.mdpi.com/article/10.3390/nu15214684/s1, CONSORT 2010 checklist of information to include when reporting a randomized trial.
